# MicroRNA Response Elements-Mediated miRNA-miRNA Interactions in Prostate Cancer

**DOI:** 10.1155/2012/839837

**Published:** 2012-11-04

**Authors:** Mohammed Alshalalfa

**Affiliations:** ^1^Department of Computer Science, University of Calgary, 2500 University Dr. NW, Calgary, AB, Canada T2N 1N4; ^2^Biotechnology Research Center, Palestine Polytechnic University, Hebron, Palestine

## Abstract

The cell is a highly organized system of interacting molecules including proteins, mRNAs, and miRNAs. Analyzing the cell from a systems perspective by integrating different types of data helps revealing the complexity of diseases. Although there is emerging evidence that microRNAs have a functional role in cancer, the role of microRNAs in mediating cancer progression and metastasis remains not fully explored. As the amount of available miRNA and mRNA gene expression data grows, more systematic methods combining gene expression and biological networks become necessary to explore miRNA function. In this work I integrated functional miRNA-target interactions with mRNA and miRNA expression to infer mRNA-mediated miRNA-miRNA interactions. The inferred network represents miRNA modulation through common targets. The network is used to characterize the functional role of microRNA response element (MRE) to mediate interactions between miRNAs targeting the MRE. Results revealed that miRNA-1 is a key player in regulating prostate cancer progression. 11 miRNAs were identified as diagnostic and prognostic biomarkers that act as tumor suppressor miRNAs. This work demonstrates the utility of a network analysis as opposed to differential expression to find important miRNAs that regulate prostate cancer.

## 1. Introduction

MicroRNAs (miRNAs) are small (18–24) nucleotide long noncoding RNAs that play a major regulatory role in a broad range of biological processes and complex diseases. Since the discovery of microRNAs [1], they emerged as a new layer of gene regulation that dramatically influence genes by binding to its 3′UTR and inactivate it by promoting its degradation or translational repression [2]. Computational predictions estimated that there are around 1700 miRNAs in human and each targets hundreds of mRNAs and over 50% of the human protein coding genes are regulated by miRNAs [3]. The area of miRNA genetics has rapidly expanded from identifying miRNAs to exploring their function and their potential as therapeutic options. Several studies have demonstrated that miRNAs are key players in the initiation and progression of cancer including prostate cancer and they act as oncogenes and tumor suppressors [4–6]. Examination of prostate tumor miRNA expression has revealed widespread dysregulation of miRNAs in primary and metastatic compared with normal prostate tissue [7]. Profiling miRNAs in various types of cancer provided evidence that miRNAs are diagnostic and prognostic biomarkers [8] that may stratify prostate tumors based on specific genetic profiles and thereby improve aspects of patient management such as staging and treatment [9, 10].

A big body of research has been focusing on identifying the functional association between miRNAs and mRNA targets. Several factors affect the association between miRNAs and targets. The first is the degree of complementarity between the miRNA and the target [11]. Several computational methods have been proposed to identify miRNAs sequence and their target based complementary sequence and thermodynamics stability with mRNA target [11, 12]. The problem with computational predictions is that they are false positive prone methods and they are not functional. The second factor is the functional association between miRNAs and targets. Since miRNA promote target degradation, a negative correlation between a miRNA and its targets is anticipated [13, 14]. Considerable body of research has concentrated on the area of integrating sequence-based miRNA target prediction and expression data to identify functional miRNA-target interactions and find miRNA-target modules. Several tools and algorithms have been developed in this respect. GenMiR++ and GenMiR3 [15] are two such tools; GenMiR3 is the modified version of GenMiR++; it combines the results of miRNA target prediction programs like TargetScanS with paired miRNA-mRNA expression data. The algorithm scores each miRNA-mRNA pair by a Bayesian approach. It evaluates whether the expression of the miRNA explains the expression level of mRNA. Target pairs get high score when miRNA or mRNA is highly expressed while the other molecule is downregulated. The third factor is endogenous RNA competition. Recently, several studies have characterized the power of miRNAs as a communication language between noncoding RNAs [16, 17]. Considerable body of research has emerged to characterize the function of noncoding RNA and the regulatory function of coding mRNAs. Results revealed that the MRE of RNAs is an important part of RNA that can play a key role in gene regulation by competing for miRNA. RNAs (coding or noncoding) that share similar MRE showed an ability modulate each other by buffering targeting miRNAs and compete for miRNA and thus influence its availability [16, 17].

Gene expression techniques are witnessing a revolution in the last decade that lead to produce very large amount of high throughput gene expression data to study cellular systems, but the drawback of these techniques is that they study individual components of the system. A major challenge in systems biomedicine is not only understanding the function of individual elements in the system, but rather understanding the function of elements as a system. There is a big gap between the biological techniques to generate high-throughput data and computational biologists who use this data to build models to explain the data. Thus a more statistical and systematic methods integrating gene expression with biological networks to have high-level understanding of biological systems function is needed. miRNA and mRNAs are two cellular molecules that interact together and regulate each other's expression. miRNAs regulate mRNAs expression by downregulating them; on the other hand, mRNA regulates miRNA by modulating its availability. Thus a new layer of gene regulation has emerged that affect mRNA expression based on miRNA language. However, how mRNA can influence and play the modulator role in miRNA regulation has not been investigated.

This study focuses on the role of MRE or 3′UTR in mediating the interactions among miRNAs. I hypothesize that MRE can modulate miRNA-miRNA interactions activity and that miRNAs can influence each other through MREs. Sponge modulators include both messenger RNAs (mRNAs) and noncoding RNAs, which contains multiple miR-binding sites for distinct miRNAs are key player in modulating miRNA availability. Depending on their expression levels and on the total number of functional miR-binding sites that they harbour, sponge modulators can decrease the number of free miR molecules available to repress other functional targets. When modulators expression is high, miRNAs targeting them will get decreased. miRNAs that share a modulator and have similar expression profile given the expression of the modulator are anticipated to be functionally related and form a posttranscriptional regulatory network. In this work a 3′UTR-mediated miRNA-miRNA in prostate cancer is constructed to identify key miRNA players that influence each other.

## 2. Materials and Methods

### 2.1. miRNA Targets Interactions

Human miRNA target predictions for miRNA with conserved 3′UTR were taken from TargetScan 5.1 [11] (PredNet), and experimentally validated miRNAs and their targets were taken from mirTarBase [8] and miRecord [20]. I used the union of mirTarBase and miRecord as a source of experimentally validated miRNA-target interactions (ExpNet). PredNet and ExpNet will constitute the miRNA-target interaction networks that will be used to associate miRNAs with their targets.

### 2.2. Expression Datasets

MRNA and miRNA expression data from the MSKCC Prostate Oncogenome Project, that is, available at the Gene Expression Omnibus (GEO accession number: GSE21032) [21], was used in this study. The data contains expression levels of 26443 genes across 179 samples (131 primary cancer, 19 metastatic, and 29 normal samples), and expression of 370 miRNAs across 140 samples. The expression data of 139 samples with both mRNA and miRNA data for our analysis was used in this study. I also used localized prostate cancer miRNA expression data from independent prostate patient cohort (GSE23022 [22]) to further validate the predicted function of miRNAs. Taylor data was used to build the network of miRNAs and GSE23022 data was used to further characterize the diagnostic role of the network results.

### 2.3. MRE-Mediated miRNA-miRNA Network Construction

**Figure 1:**
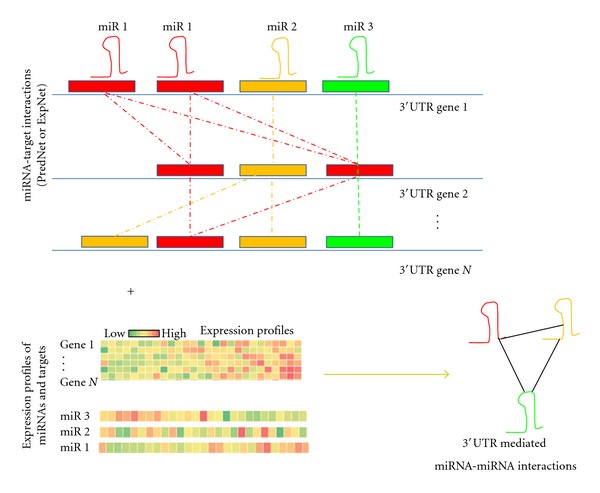
Overview of MRE-mediated miRNA-miRNA network construction. miRNA-miRNA interaction network was constructed by combing miRNA-target networks and expression profiles of both miRNAs and targets. I considered competition between miRNAs for common targets to construct miRNA-miRNA network. miRNAs that target same 3′UTR or MRE and are conditionally dependent on target are anticipated to be functionally associated.

Here I describe the mathematical model used to build mRNA-mediated miRNA-miRNA interaction network as illustrated in Figure 1. I hypothesize that the expression of mRNA(m) can modulate interactions between miRNAs (miR1, miR2) that have miR-binding site in the MRE of the mRNA (m). The model requires miRNA-target interaction data (PredNet or ExpNet) and expression profiles of miRNAs and targets from the same sample set. To find the mRNA(m)-mediated miRNA-(miR1-) miRNA(miR2) interactions, I used conditional mutual information as described in [17]. Mutual information between miR1 and miR2 is calculated as

(1) $$\begin{array}{c} \text{M}{\text{I}}_{\text{miR}1,\text{miR}2}=\sum_{d\in \text{miR}1}\sum_{\;\;r\in \text{miR}2}p\left(d,r\right)\log\frac{p\left(d,r\right)}{p\left(d\right)p\left(r\right)}, \end{array}$$

where *p*(*d*, *r*) is the joint probability density function (pdf) of miR1 and miR2, and *P*(*d*) and *p*(*r*) are the marginal pdf's of miR1 and miR2, respectively. The modulation effect of mRNA (m) on miR1 and miR2 is calculated as

(2) $$\begin{array}{c} \Delta \text{M}{\text{I}}_{\text{miR}1,\text{miR}2/\text{m}}=\left|\text{MI}\left(\text{miR}1,\text{miR}2\right)-\text{MI}\left(\text{miR}1,\frac{\text{miR}2}{m}\right)\right|. \end{array}$$

The significance (*P*  value  (miR1, miR2/*m*)) for each interaction (miR1, miR2) given a mRNA (*m*) was calculated by permutating (shuffling) the expression values of *m* across the 139 samples 1000 times and then comparing the observed ΔMI value against the expected ΔMI values from the permutation distribution. Since miRNAs target multiple mRNAs, I obtained multiple *P* values for each miRNA-miR interaction given a mRNA and then find the final significance *P* value across all mRNA targets by converting individual *P* values *p*_*k*_ for each miRNA-miR to a *X* ^2^ test statistics using Fisher's method, where *X* ^2^ = −2∑_*k*=1_^*N*^ln⁡(*p*_*k*_), where *N* is the total number of targets for miRNA. This method is very similar to the miRNA mediated mRNA interactions constructed by Sumaizin et al. [17] but the major difference is that they consider miRNAs as modulators and I consider mRNAs as modulators.

Computing the mutual information between all miRNAs pairs given all possible mRNAs is very time consuming. Thus I used prior knowledge from miRNA-target interactions as a base to predict mRNA mediated miRNA-miRNA interactions. For each miRNA from miRNA-target interaction in PredNet or ExpNet and its targets, I calculated ΔMI_miRNA,miR/target_ across all miRNAs (miR) in Taylor dataset and obtained a *P* value by shuffling the expression of target across the 139 samples. As a result I obtained a *P* value for each miRNA-miR interaction given a target. The final step was to generate one *P* value for the miRNA-miR interactions that depend on different targets. The *P* values were converted using Fisher's method. Only significant (*P* < *e* ^−5^) were considered to build the miRNA-miRNA interactions network. 

## 3. Results

### 3.1. MRE-Mediated miRNA-miRNA Interactions

**Figure 2:**
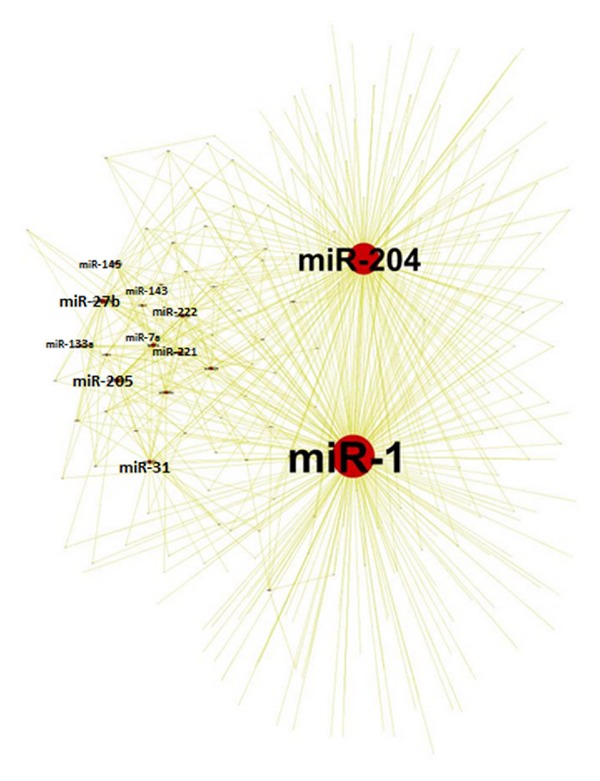
MRE-mediated miRNA-miRNA interactions network using ExpNet. miRNA-miRNA interactions using ExpNet show a list of 11 miRNAs that are highly connected. The network shows 243 miRNA linked with 528 link. miRNA-1 and miRNA-204 are hub miRNAs that are linked to more than 50% of the miRNAs. The size of the miRNA node is proportional to the miRNA connectivity. Cytoscape was used for network visualization [18].

Genome-wide inference of MRE modulators identified a miRNA-miRNA posttranscriptional regulatory network. I modelled the network graphically, with miRNAs represented as nodes and their MRE-mediated interactions as undirected edges. This network represents an indirect regulatory effect between miRNAs. A miRNA can influence the expression of its miRNA partners by regulating their common target and thus modulate the partner's miRNA activity. I first used ExpNet data to construct the miRNA-miRNA network.  Figure 2 shows 528 interactions among 243 miRNAs. The miRNAs in the network have an average of 20 interactions ranging from 1 to 190. This suggests that, on average, there are 20 targets that mediate interactions between miRNA pairs.

**Figure 3:**
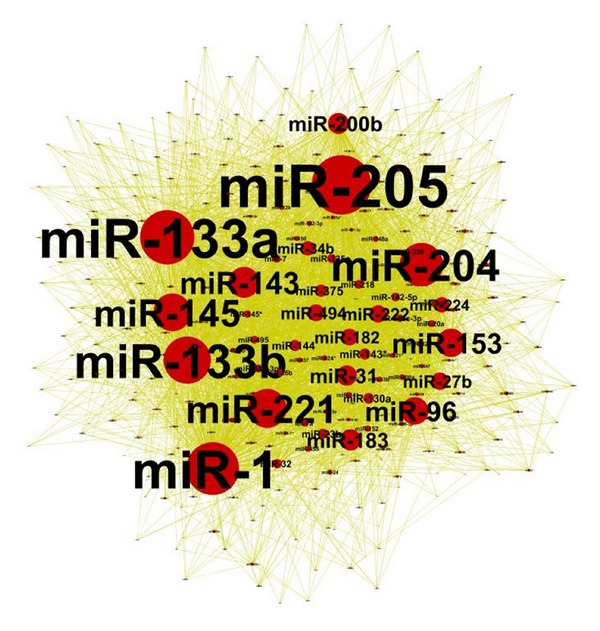
MRE-mediated miRNA-miRNA interactions network using PredNet. 3753 miRNA-miRNA interactions among 345 miRNA was constructed using PredNet. A list of 16 miRNAs that are highly connected. miRNA-1, miR-133a, miR-133b, miR-221, miR-145, and miRNA-205 are hub miRNAs that are linked to more than 50% of the miRNAs. Cytoscape was used for network visualization. It is worth pointing out the difference between Figures 2 and 3 is that Figure 2 uses ExpNet miRNA-target interactions and Figure 3 uses PredNet miRNA-target interactions.

The overall regulatory effect on a node depends on several variables, including the number of mRNAs that harbour a binding site for the node, the number of distinct binding sites on a mRNA, and the expression level of the modulator mRNA. 11 miRNAs are more connected than others. The network revealed that miNRA-1 and miRNA-204 are hub miRNAs in the network with connection to more than 70% of the miRNAs in the network. Additional miRNAs like miR-205, miR-27b, miR-31, miR-222, miR-221, miR-133a, miR-143, let-7a, and miR-145 was shown also to be highly connected to other miRNAs. I will refer to this set of miRNAs as the 11 miRNAs. I further applied the method on PredNet and showed that the 11 miRNAs are hub nodes (Figure 3). This suggests that the resulting network is not biased to the miRNA-target interactions used.

I then asked if the resulting MRE-mediated miRNA-miRNA network is biased to the number of mRNA targets that miRNAs share. So I constructed a miRNA-miRNA network (Figure  S1) based only on the common mRNA targets. Interactions in this network represent how much the two partner miRNAs share common targets. Surprisingly, none of the hub miRNAs in Figure 2 showed any significance. This means that the interactions among the 11 miRNAs and the high connectivity of the 11 miRNAs is not due the large number of common binding sites between miRNAs. I then constructed another miRNA-miRNA based on expression correlation (Figure  S2) and found interesting results. The identified hub miRNAs are highly correlated with each other, but not correlated with other miRNAs in the genome. Several miRNAs like miR-1224-3p and miR-937 was shown to be correlated with most of the miRNAs but not with our identified list of miRNAs. This result indicates that our hub miRNAs are correlated in subset of samples and not all of them.

Further an MRE-mediated miRNA-miRNA network was constructed using only expression data from primary prostate samples (98). The purpose of this network is to assess if the MRE-mediated miRNAs network is biased to the primary cancer samples that constitute large portion of the samples used in the study. Also it helps to reveal miRNAs that may play a role in subtyping primary cancer. Interestingly miR-1, miR-155, and miR-16 were found to be hub miRNA. This could shed light on the role of miRNAs in different stages of prostate cancer progression.

### 3.2. Tumor Suppressor miRNAs Network in Prostate Cancer

To find the robustness of the interactions between the 11 miRNAs, PredNet was used to construct the MRE-mediated miRNA-miRNA interactions of 345 miRNA nodes and 3753 edges. A more stringent cutoff was then used to reduce the network to 70 miRNA nodes connected by 128 edges. Both resulting networks support that the 11 miRNAs are significantly associated and miRNA-1 which is the master regulator of miRNA interactions. This result indicates that the 11 miRNAs are regulating each other and they have similar mode of actions. Several miRNAs among the 11 miRNA (miRNA-1, miRNA-145, miRNA-143) have been characterized as tumor suppressors in prostate cancer [3, 6]. Thus our network results suggest that the other miRNAs may act as a network of tumor suppressors.

### 3.3. Functional Analysis of Key 11 miRNAs

**Figure 4:**
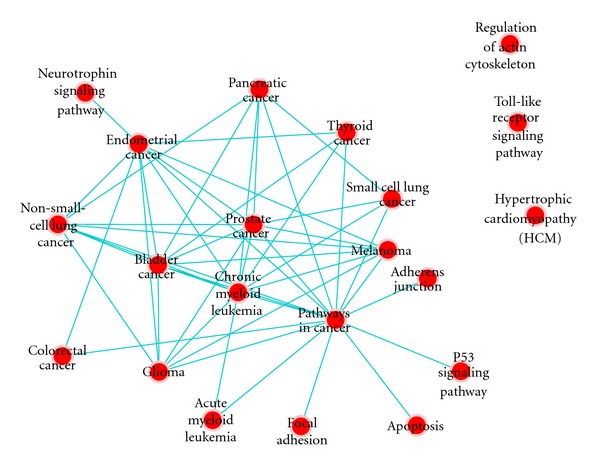
Pathway enrichment analysis of the 450 target genes of the 11 miRNA using Enrichment Map [19]. 450 genes targeted by the 11 miRNA were identified using ExpNet. I used DAVID online tool to identify enriched pathways of the 450 genes using Enrichment Map [19]. Results showed that the target genes are enriched with multiple cancer pathways including prostate, thyroid, and pancreatic cancer pathways.

**Figure 5:**
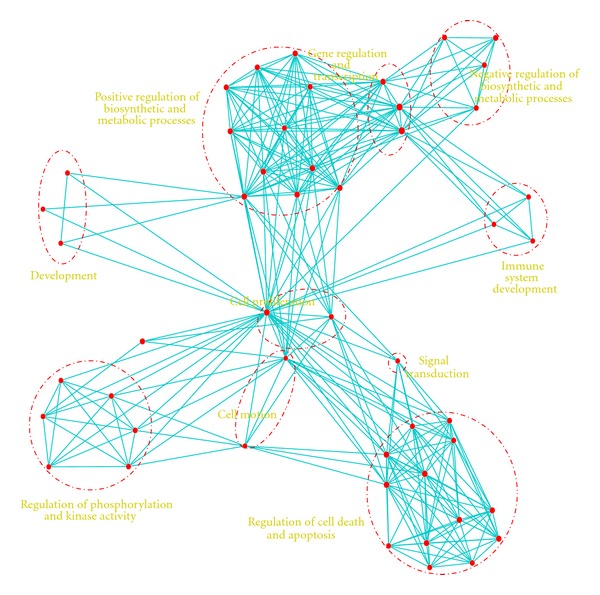
Biological processes enrichment analysis of the 450 target genes of the 11 miRNA using Enrichment Map [19]. Analyzing the biological processes enriched in the miRNA target genes using DAVID tool showed that targets are enriched with several biological processes like cell proliferation, cell death, and biosynthetic metabolism. Enrichment Map [19] was used for visualizing the network of biological processes.

Here I asked if the 11 key miRNAs (hub miRNAs) have any functional role in prostate cancer. I extracted the target genes of the 11 key miRNAs from experimentally verified miRNA-target interactions (ExpNet) and characterized their function using DAVID online tool (http://david.abcc.ncifcrf.gov/). 462 genes are targeted by the 11 miRNAs, 240 of them are targeted by miRNA-1. The function of the 462 genes was characterized by analyzing the biological pathways they are involved in and the biological processes they are part of in addition to biological terms associated with them. Target genes are associated with phosphoprotein (1.1 × *e* ^−21^), proto-oncogene (2.6 × *e* ^−13^), disease mutation (5.2 × *e* ^−8^), acetylation (6.7 × *e* ^−7^), actin-binding (1.2 × *e* ^−6^), and apoptosis (8.2 × *e* ^−6^). Analyzing the pathways revealed strong correlation between the target genes and several types of cancer including prostate, melanoma, thyroid, pancreatic cancers (Figure 4). Target genes also are involved in several biological processes like cell proliferation, cell motion, regulation of cell death, regulation of biosynthetic, and metabolic processes and kinase activity (Figure 5). All these enrichment analysis support that the 11 miRNAs play key role in prostate cancer by targeting genes from multiple biological processes. Though the enriched pathways are not prostate specific, they show that the 11 miRNAs target core pathways and mainly phosphorylation signaling pathways.

### 3.4. Diagnostic and Prognostic Relevance of Key miRNAs

**Figure 6:**
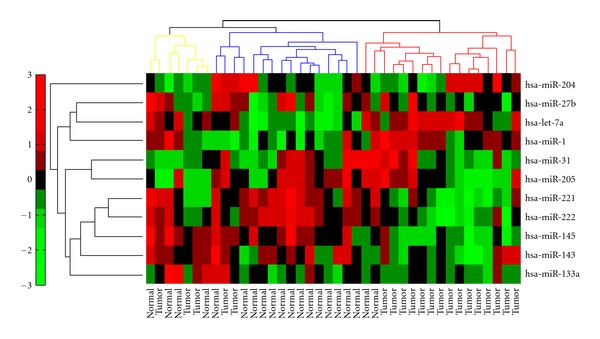
Heatmap of the 11 miRNA in GES23022 prostate data. Heatmap of the 11 miRNAs shows that the 11 miRNAs are effective to group tumor samples. Clustering the samples using *k*-means revealed three groups, tumor, normal, and mixed cluster.

**Figure 7:**
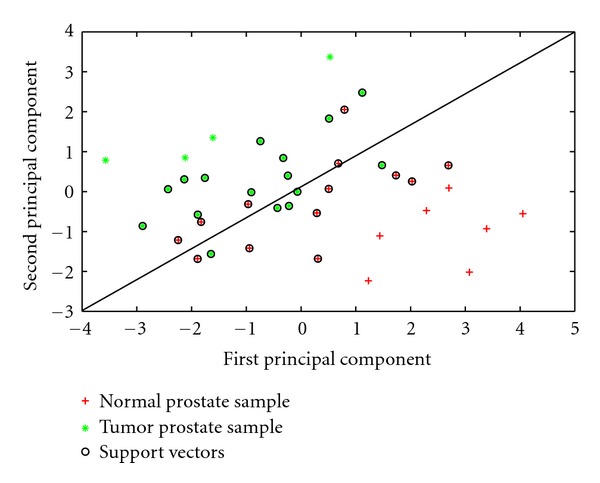
SVM classification of samples in GSE23022 across the first two PCAs. I first identified the first two principal components (PCAs) and then use SVM to classify samples based on the first two components. Results show that normal and tumors samples are separated with some misclassification at the boundary of the support vectors.

The diagnostic and prognostic power of the 11 miRNAs was further characterized using independent prostate expression dataset. The expression of the 11 miRNAs was extracted from GSE23022 and analyzed the power of the 11 miRNA in discriminating tumor samples from normal samples using three methods. Hierarchical clustering was used to group patients based on the expression level of the 11 miRNAs (Figure 6). The heatmap clearly shows three distinct groups: tumor, normal, and a mixed group. Principal component analysis showed that normal and tumor samples are distinguishable using the first three principal components (Figure 7). Finally, support vector machine classified the samples (tumor versus normal) using the expression of the 11 miRNAs with 85%. To find the significance of this classification, I randomly generated 1000 lists of 11 miRNAs and calculated the average accuracy (50.7%) *P* value (0).

**Figure 8:**
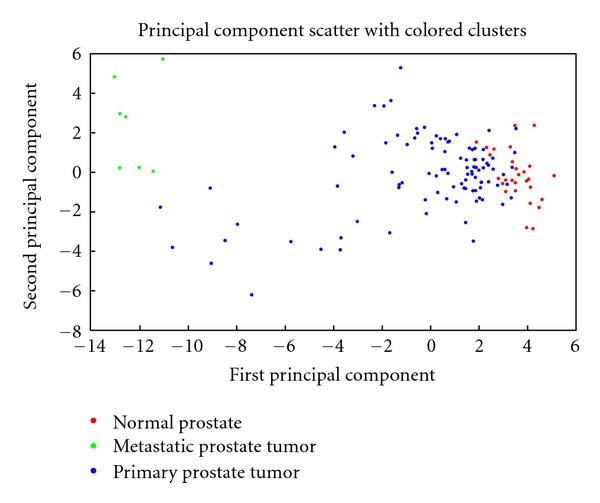
Prostate samples across the first two PCAs using Taylor miRNA expression data. I first identified the first two principal components (PCAs) using Taylor data that has normal, primary, and metastasis samples. Results show that metastasis samples are well separated from normal and primary samples across the first component.

Taylor data was used to assess the power of the 11 miRNA to discriminate primary from normal samples (88% versus 77% (random)) (Figure S4). I compared this result with the 11 most downregulated genes identified using significant analysis of microarray (SAM) [23] and found that 5 (miR-221, 222, 145, 133a, 143)out of the key miRNAs are among the 11 most downregulated genes. The top 11 upregulated genes were able to classify cancer from normal samples with 90% and the 11 downregulated genes are able to classify the samples with 86%. This indicates that the 11 hub miRNAs are good diagnostic biomarkers. I then analyzed the power of the 11 miRNAs to discriminate metastatic samples from primary cancer using Taylor gene expression data. Results showed that the 11 miRNAs significantly discriminate primary from metastatic samples with 99.1% using SVM. Using random list of 11 miRNAs resulted in an average of 88.8% *P* value (0). Principal component analysis showed that metastatic samples are clearly separated from primary and normal (Figure 8); however, some primary samples are very close to normal samples. One reason could be because the primary samples are still in early stages of cancer and due to the heterogeneity of the primary cancer. This indicates that these 11 miRNAs are important molecules in prostate cancer initiation and progression.

**Figure 9:**
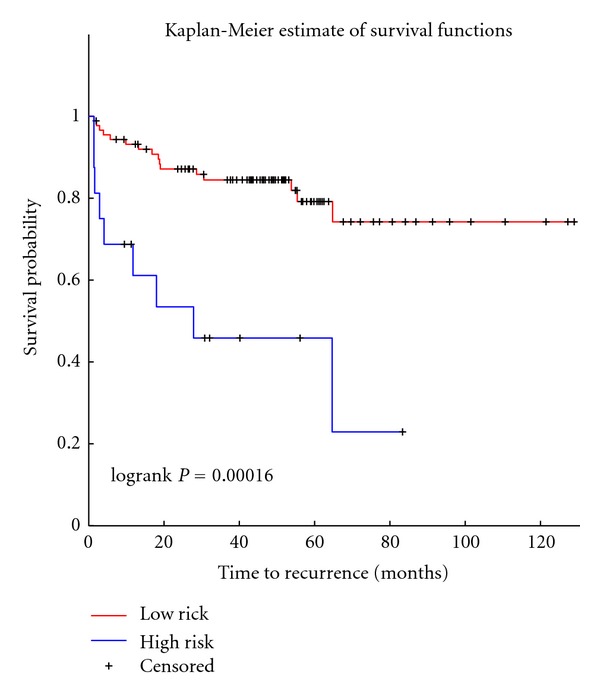
Kaplan Meier plot of the prognostic power of the 11 miRNAs. We characterized the prognostic power of the 11 miRNAs by extracting their expression from Taylor data and group samples based on their expression into two groups. The two groups showed a very significant separation between high-risk and low-risk patients. This indicates that the 11 miRNA can act as therapeutic targets for prostate cancer treatment.

To analyze the prognostic power of the 11 miRNAs, I extracted their expression from Taylor data and used cox regression model to find association between miRNAs expression and cancer recurrence. Clustering the samples using *k*-means based on the 11 miRNAs into two groups showed that the two groups have significant different outcome (HR: 4.9, 95%CI (2.15–11.19), *P* = 0.00016) (Figure 9). Using univariate cox regression revealed that all the 11 miRNAs are associated with outcome; low expression level of the 11 miRNA is associated with aggressive cancer. Using univariate regression model showed that miR-1 is the most significant miRNA associated with outcome. I then analyzed the differential expression profiles of the 11 miRNAs in aggressive cancer versus nonaggressive (based on Taylor groups) and found that all of them are significantly downregulated in aggressive cancer (*P* less that 1 × 10^−5^).  Table 1 shows the hazard ratio, multivariate regression coefficients, and the differential expression power using fold change and SAM analysis.

## 4. Discussion

The applications of systems biology to understand complex disease driven by the fact that complex diseases like cancer are attributed to dysregulation of multiple components of the cellular system [24]. Prostate cancer is the most widely spread cancer in male in western countries. One of the challenging in studying prostate cancer is the heterogeneity of the system. Several genes are attributed to initiate and develop prostate cancer, in addition to role of miRNAs in initiating and progressing prostate cancer [4]. Several miRNAs profiling studies have been conducted to identify miRNAs that are differentially expressed in tumor versus normal tissues [10]. Identifying prognostic miRNAs that can help to predict patient outcome or the stage of disease is another important aspect to understand diseases progression. Identifying miRNA-mRNA function modules is another important task in miRNA genetics. One of the least studied factors affect the functionality of miRNAs is competing for target. Recent study showed that targets that compete for miRNAs pose a regulatory effect on each other by limiting the availability of miRNA [16, 17]. Using this notion, Sumazin et al. [17] generated a miRNA-mediated network among RNA molecules. Here it is worth mentioning that miRNAs mediate all RNA molecules that harbour a binding site for the miRNA. This study motivated us to analyze the systematic function of miRNAs in prostate cancer by analyzing the influence of each miRNA on the other miRNAs through the target. miRNAs that share MRE of several targets and their expression conditionally dependent on the target are anticipated to regulate each other.

In this work I analyze the functional role of miRNAs in prostate cancer by integrating expression data of targets and miRNAs and miRNA-target networks. Several studies that integrated expression data with miRNA-target networks lead to identifying miRNA-target modules that may play a role in prostate cancer [14]. However, in this work I integrated expression data using conditional mutual information to assess the conditional dependence between pairs of miRNA and their common target(s). miRNAs modulate each other through their common targets that affect miRNA availability. The association between miRNAs depends on the number of common targets and the significance of the conditional dependence on the target.

One of the challenges I faced in this study is constructing the miRNA-miRNA interaction network using all possible targets as mediators that is computationally very expensive. To reduce computational cost, I started with the miRNA-target network and we only computed conditional dependency between one miRNA and the rest of miRNAs given the expression of the targets. I used both experimentally verified and computationally predicted miRNA-target interactions to identify the miRNA-miRNA networks. Both networks showed that miRNA-1 is a hub miRNA in both networks. This indicates that it has regulatory effect over other miRNAs through its targets. Based on the two networks (Figures 2 and 3), 11 miRNAs were identified as hub miRNAs and further analyzed their function and prognostic role. Analyzing their function showed that they play a role in several biological processes including cell proimmigration, cell death, and metabolic biosynthesis (Figure 5). Analyzing their prognostic role revealed that the 11 miRNAs act as diagnostic and prognostic biomarkers. The low expression of the 11 miRNAs showed to be associated with cancer recurrence (Figure 9). Several miRNAs among the 11 miRNas are already in clinical trials (miR-16, miR-222, miR-221) [3]. Here it is worth mentioning that the 11 hub miRNAs are not the top differentially downregulated miRNAs but they are powerful diagnostic biomarkers.

The results in this work caught the attention to the significance of miR-1. Therefore, I further investigated its role in prostate cancer and argue that it is the guardian of the miRNA-mediated gene expression control. microRNA-1(miR-1) is reported to be one of the most consistently downregulated microRNAs in human prostate tumors [25]. Recent study showed that miR-1 is further reduced in distant metastasis tumors and is a candidate predictor of disease recurrence. miR-1 is encoded by the miR-1-133 cluster which has two copies (at 18q11 and 20q13) in the human genome producing identical mature miR sequences for miR-1 and miR-133. It was recently reported that miR-1, miR-133, and miR-206, which is a functional homolog of miR-1, are among the most frequently downregulated miRs in solid human cancers. Recent study reexpressed miR-1 in human prostate cancer cell lines and their results revealed that miR-1 is a novel candidate marker for disease recurrence in prostate cancer and exhibits a tumor suppressor activity that affects multiple pathways, leading to higher order chromosomal and epigenetic alterations globally similar to those of histone deacetylase inhibitors. Our results found that miRNA-1 targets 240 genes from ExpNet and 527 in PredNet. Both lists showed that they are enriched with phosphoproteins (5.3 × *e* ^−6^) and acetylation proteins (3.7 × *e* ^−7^). 3′UTR-mediated miRNA interactions show consistent results that miRNA-1 is a hub miRNA using different miRNA-target interactions with different cutoff values (Figures 2 and 3). I found that miRNA-1 is hub in primary prostate cancer network and a hub in the other miRNA-miRNA networks revealed that miRNA-1 is a key regulator of genes and a master coordinator of other miRNAs. Epigenetic analysis showed that promoter hypermethylation may be the reason behind the reduced expression of miRNA1-133 cluster including miRNA-1 [25].

Next I asked if the miRNA interactions are biased toward other factors that may influence the association among miRNAs. First, correlation between miRNAs was shown to influence the interactions among the 11 miRNAs. I found that the 11 miRNAs are correlated but they are not correlated with other miRNAs, which indicates that these miRNAs have something common between them and not with other miRNAs. The second factor is the number of common targets that might influence the network. So I calculated the association between miRNAs based on the number of common targets between them and found that the 11 miRNAs are not significantly connected and they are not among the hub genes. This indicates that the number of common targets between miRNAs did not influence the interactions between miRNAs.

Lastly, the expression of primary prostate samples was used to identify the miRNA-miRNA interactions based on expression of primary cancer samples alone. Interestingly, I found that miR-1 is still the most connected miRNA and other miRNAs (miR-155, miR-16) are hubs. This indicates that these miRNAs play a significant role in cancer initiation and not metastasis.

## 5. Conclusion

As the field of miRNA continues to grow, a deeper understanding of miRNA expression, function, and control in prostate cancer will influence the development of miRNA-based therapeutics. In this work I showed that miRNA-1 is a key player in regulating gene expression and has high influence on other miRNAs. 11 miRNAs are identified as a network of tumor suppressors that have prognostic role in cancer recurrence.

## Supplementary Material

The supplementary file provides more comparative analysis to compare the MRE mediated miRNA-miRNA interactions produced in this study with correlated miRNA network from miRNA expression and miRNA interactions based on number of common gene targets. Results reveals that the MRE-mediated miRNA-miRNA interaction network is not biased to the correlation between the miRNAs nor it is biased to the number of common target the miRNAs share. This suggests that there is another principle that governs the produced miRNA interaction network. The robustness of the MRE-mediated miRNA-miRNA network was further assessed by using experimentally validated miRNA-target interactions instead of computationally predicted interactions. The results supported the initially observed results that suggest that miRNA-1 is the master regulator of prostate cancer. The diagnostic power of the 11 miRNAs is tested in Taylor prostate data to support that the 11 miRNAs are robust diagnostic biomarkers.

## Figures and Tables

**Table 1:** Diagnostic and prognostic characteristics of the 11 miRNAs from Taylor data.

	HR (95% CI)	Cox multivariate regression coefficient	Fold change (cancer/normal)	SAM *q*-value
hsa-let-7a	0.77 (0.18–3.26)	−0.25	0.88	2.3
hsa-miR-1	0.30 (0.09–0.9)	−1.17	0.64	0
hsa-miR-133a	1.23 (0.41–3.67)	0.20	0.43	0
hsa-miR-143	1.67 (0.53–5.19)	0.51	0.49	0
hsa-miR-145	1.72 (0.31–9.6)	0.54	0.45	0
hsa-miR-204	1.34 (0.68–2.66)	0.29	0.48	0
hsa-miR-205	1.03 (0.8–1.34)	0.03	0.22	0
hsa-miR-27b	2.85 (0.77–10.5)	1.05	0.63	0
hsa-miR-221	0.29 (0.05–1.53)	−1.22	0.42	0
hsa-miR-222	1.23 (0.23–6.4)	0.2	0.35	0
hsa-miR-31	0.63 (0.31–1.2)	−0.45	0.39	0
