# Supplementary material for: MicroRNA Response Elements-Mediated miRNA-miRNA Interactions in Prostate Cancer
Source: Adv Bioinformatics. 2012 Nov 4;2012:839837. doi: 10.1155/2012/839837 (PMC3502784; doi:10.1155/2012/839837)
Supplement: Supplementary file 1 — The supplementary file provides more comparative analysis to compare the MRE mediated miRNA-miRNA interactions produced in this study with correlated miRNA network from miRNA expression and miRNA interactions based on number of common gene targets. Results reveals that the MRE-mediated miRNA-miRNA interaction network is not biased to the correlation between the miRNAs nor it is biased to the number of common target the miRNAs share. This suggests that there is another principle that governs the produced miRNA interaction network. The robustness of the MRE-mediated miRNA-miRNA network was further assessed by using experimentally validated miRNA-target interactions instead of computationally predicted interactions. The results supported the initially observed results that suggest that miRNA-1 is the master regulator of prostate cancer. The diagnostic power of the 11 miRNAs is tested in Taylor prostate data to support that the 11 miRNAs are robust diagnostic biomarkers. [file 839837.f1.docx]

MicroRNA Response Elements-Mediated miRNA-miRNA Interactions in Prostate Cancer

Mohammed Alshalalfa

Supplementary Figures


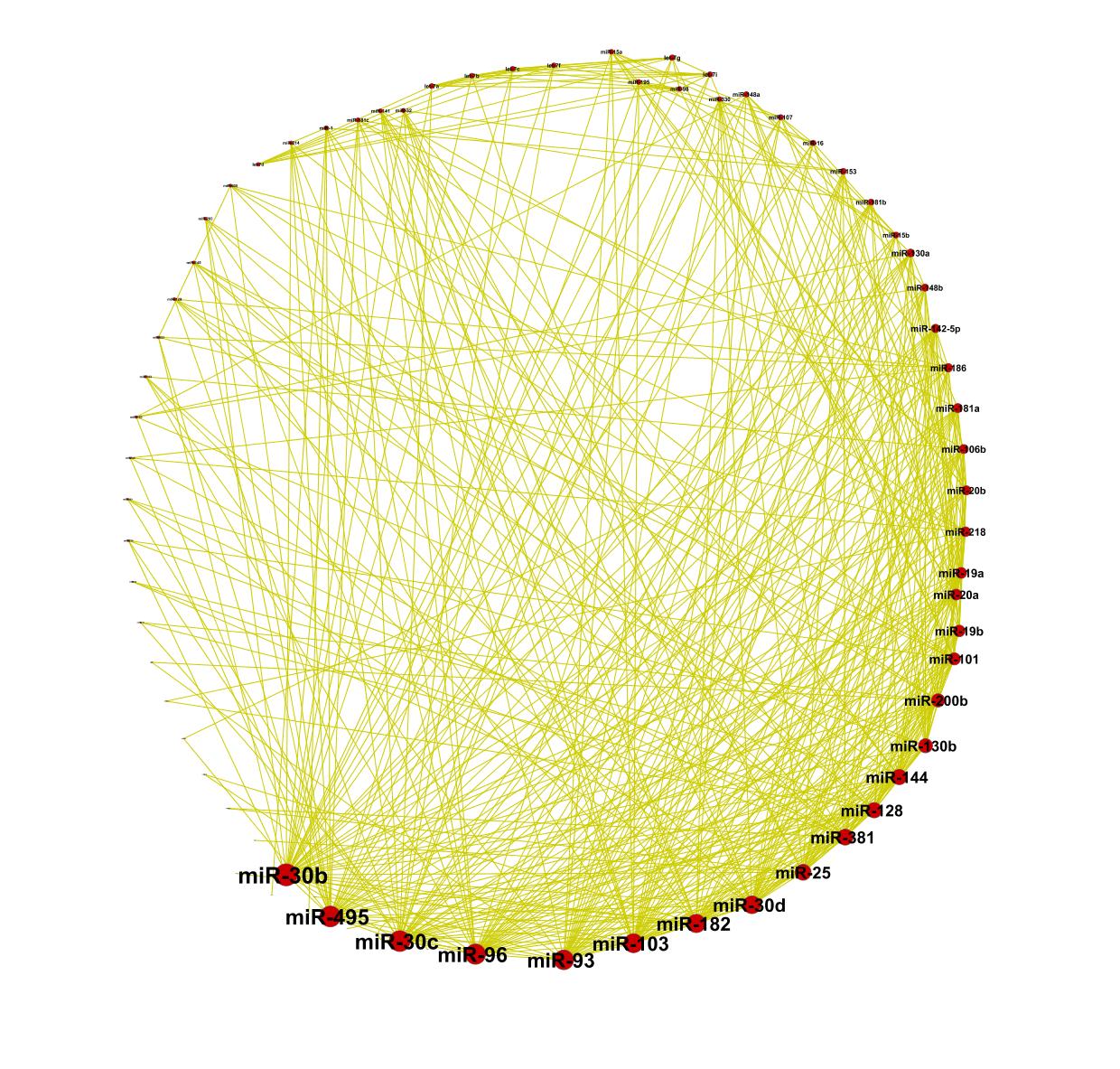


Figure S1: **miRNA binding site based miRNA-miRNA interactions network using PredNet.** The figure shows interactions between miRNAs based on the number of common targets.


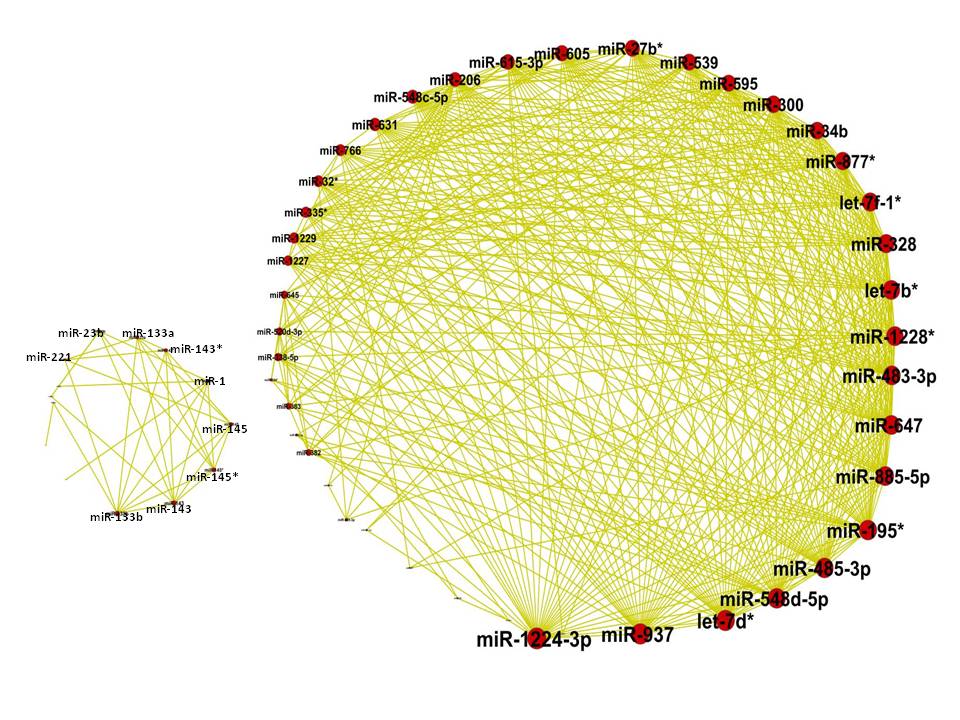


Figure S2: **miRNA expression correlation based miRNA-miRNA interactions network**. I used mutual information to find correlation between miRNAs expression profiles across all samples using Taylor data . The figure shows two clusters of correlated miRNAs. Interestingly our 11 miRNAs are connected to each other but not connected to the other miRNAs.


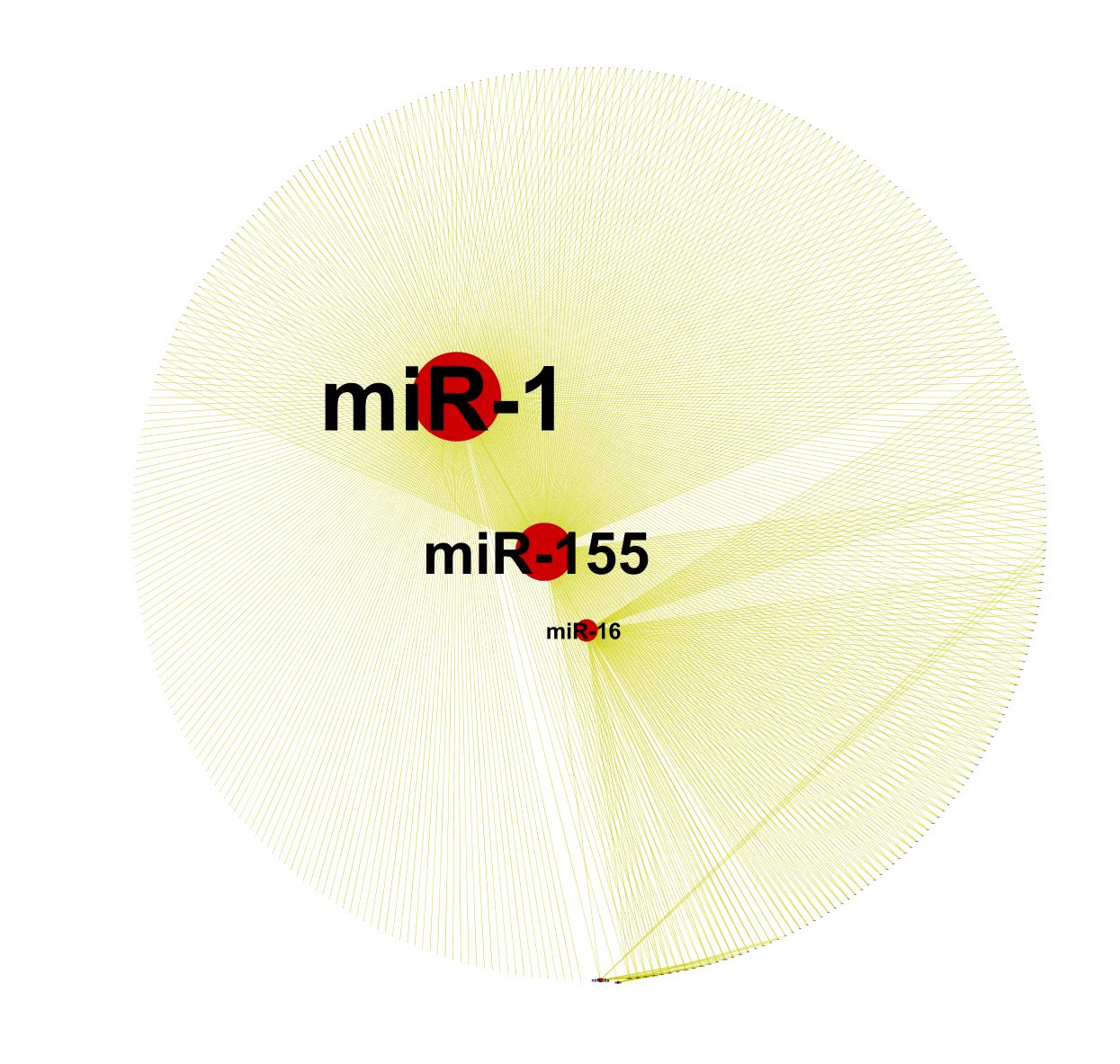


**Figure S3: MRE-mediated miRNA-miRNA interactions network using ExpNet miRNA-target network and primary cancer gene expression data**. The network shows MRE-mediated miRNA-miRNA interactions using only primary cancer data and ExpNet as miRNA-target network. The figure shows that miRNA-1 , miR-155 and miR-16 as hub miRNAs that are linked to more than 70 %of miRNAs. This indicates that these three miRNAs may play a role in prostate cancer initiation and development.


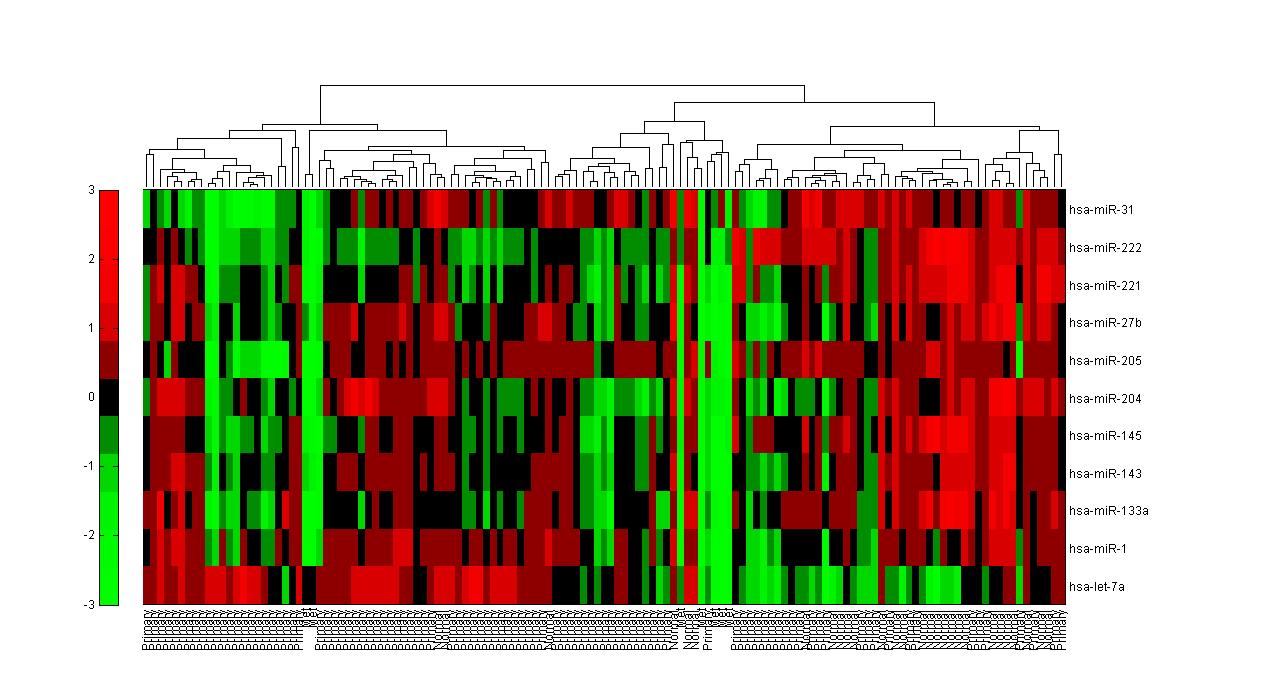


**Figure S4: Heatmap of the 11 miRNAs from Taylor data.** Results show that the 11 miRNAs are up-regulated in normal samples and down-regulated in primary samples. Heatmap reveals also that the 11 miRNAs are further down-regulated in metastatic samples.
